# Hydrocephalus induces dynamic spatiotemporal regulation of aquaporin-4 expression in the rat brain

**DOI:** 10.1186/1743-8454-7-20

**Published:** 2010-11-05

**Authors:** Anders D Skjolding, Ian J Rowland, Lise V Søgaard, Jeppe Praetorius, Milena Penkowa, Marianne Juhler

**Affiliations:** 1 University Clinic of Neurosurgery, Rigshospitalet, Copenhagen, Denmark; 2 Danish Research Centre for Magnetic Resonance, Copenhagen University Hospital Hvidovre, Hvidovre, Denmark; 3 The Water and Salt Research Center, Department of Anatomy, Aarhus University, Aarhus, Denmark; 4 Department of Neuroscience and Pharmacology, Faculty of Health Sciences, University of Copenhagen, Copenhagen, Denmark; 5 Dept. of Radiology, University of Wisconsin-Madison, Madison, USA

## Abstract

**Background:**

The water channel protein aquaporin-4 (AQP4) is reported to be of possible major importance for accessory cerebrospinal fluid (CSF) circulation pathways. We hypothesized that changes in AQP4 expression in specific brain regions correspond to the severity and duration of hydrocephalus.

**Methods:**

Hydrocephalus was induced in adult rats (~8 weeks) by intracisternal kaolin injection and evaluated after two days, one week and two weeks. Using magnetic resonance imaging (MRI) we quantified lateral ventricular volume, water diffusion and blood-brain barrier properties in hydrocephalic and control animals. The brains were analysed for AQP4 density by western blotting and localisation by immunohistochemistry. Double fluorescence labelling was used to study cell specific origin of AQP4.

**Results:**

Lateral ventricular volume was significantly increased over control at all time points after induction and the periventricular apparent diffusion coefficient (ADC) value significantly increased after one and two weeks of hydrocephalus. Relative AQP4 density was significantly decreased in both cortex and periventricular region after two days and normalized after one week. After two weeks, periventricular AQP4 expression was significantly increased. Relative periventricular AQP4 density was significantly correlated to lateral ventricular volume. AQP4 immunohistochemical analysis demonstrated the morphological expression pattern of AQP4 in hydrocephalus in astrocytes and ventricular ependyma. AQP4 co-localized with astrocytic glial fibrillary acidic protein (GFAP) in glia limitans. In vascular structures, AQP4 co-localized to astroglia but not to microglia or endothelial cells.

**Conclusions:**

AQP4 levels are significantly altered in a time and region dependent manner in kaolin-induced hydrocephalus. The presented data suggest that AQP4 could play an important neurodefensive role, and may be a promising future pharmaceutical target in hydrocephalus and CSF disorders.

## Background

Hydrocephalus is commonly defined as increased amount of cerebrospinal fluid (CSF) in a dilated ventricular system, and the pathophysiology of the disease is therefore strongly correlated to defects in CSF-circulation [1]. Traditionally CSF circulation has been described in a hydrodynamic perspective. "The classic bulk flow theory" explains the CSF circulation as a bulk flow of fluid from the production site at the choroid plexus, through the ventricular system and into the subarachnoid space (SAS). Here the CSF is finally absorbed into the superior sagittal sinus through the arachnoid granulations. The driving force is thought to be a net positive pressure gradient between the choroid plexus and the superior sagittal sinus to overcome the resistance in the arachnoid granulations [1]. Several authors have questioned this theory, and accessory pathways have been proposed [2-6]. The accessory pathways involve absorption sites other than the arachnoid granulations, and are thought to consist of both direct absorption through parenchyma to brain capillaries and absorption through lymphatic vessels with relation to cranial nerves [2-6]. It has been suggested that the accessory pathways predominate in the immature human brain, and that the traditional arachnoid pathway develops throughout childhood. The idea of accessory pathways has gained new interest through the observation of relatively high failure rate of neuroendoscopic ventriculostomy in neonates and infants [5]. Oi and Di Rocco [5] reviewed the ontogenesis of arachnoid granulations and "CSF Dynamics Maturation Stages" in human with adult animals and found that mice, rats and rabbits are comparable with neonates and infants, underlining the relevance of the present study.

Aquaporins (AQP) are cellular transmembrane proteins with a central pore [7]. This pore is specific to the passage of water molecules exclusively in the orthodox aquaporins, while aquaglyceroporins are permeable to water and other small uncharged molecules such as glycerol, urea and lactate [8,9]. AQP4 is the most abundant aquaporin of the brain [10,11] being expressed in astrocytic processes including their perivascular endfeet and processes of the glia limitans. AQP4 is also found in the basolateral membrane of ependymal cells [12-14]. The location of AQP4 is therefore specific to blood-tissue and tissue-CSF border, possibly facilitating fast water transport dependent on osmotic gradients between those compartments [15-17]. Limited published work in this field [15,17-19] suggests that the parenchymal CSF absorption route is highly dependent on AQP4. Expression levels of AQP4 could possibly influence resolution of interstitial hydrocephalic edema and drainage of excess ventricular CSF. In this study we hypothesized that changes in AQP4 expression in specific brain regions correspond to the severity and duration of hydrocephalus. Hence our objective was to study the spatiotemporal changes in brain AQP4 expression during experimental hydrocephalus relative to healthy physiological conditions.

## Methods

### Ethics and study design

The study was approved by the Danish Animal Experiments Inspectorate (Ministry of Justice, license number 2006/561-1169) and carried out in accordance with both European and Danish law and legislation for laboratory animal experiments. Animal welfare was protected by minimizing animal numbers, pain, suffering, and lasting harm. We used an experimental kaolin model of hydrocephalus in rat compared to a control group. Magnetic resonance imaging (MRI) was used to describe the hydrocephalic condition (ventricular size, brain water diffusion and blood brain barrier (BBB) integrity). For tissue analysis antibody-based methods (Western blotting (WB), immunohistochemistry (IHC) and immunofluorescence (IF)) were used to investigate AQP4 expression, cellular localization, and quantification during hydrocephalus.

**Figure 1:**
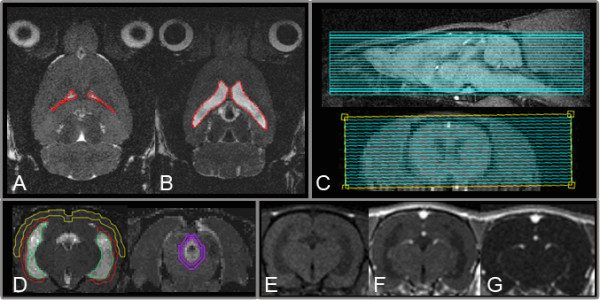
**MRI imaging**. A T2-weighted fast spin-echo sequence was used for quantification of lateral ventricular volume (A: control, B: 2W kaolin). Twenty-two contiguous T2-weighted axial slices were acquired in interleaved order, as shown in C. For quantification of water diffusion, four different regions of interest (cortex (yellow), periventricular (red), periaqueductal (purple) and CSF (green)) were drawn, using the b = 0 images, and apparent diffusion coefficient values were calculated for each ROI (D). In addition, T_1_-weighted pre (E) and post (F) contrast images were acquired. Blood brain barrier integrity was determined by subtraction of pre-contrast images from post-contrast images (G).

The study focused on predetermined regions of interest (ROIs) (Figure 1) to correlate regional AQP4 expression with imaging by using MRI. The aim was to classify the temporal expression pattern by comparing the brain AQP4 levels with brain water diffusion and lateral ventricular volume at 2 days, 1 week, and 2 weeks after the induction of hydrocephalus. As we wished to study the hydrocephalic condition, three non-hydrocephalic animals (all belonging to the two week group) were omitted from the data analysis.

### Induction of hydrocephalus

Sprague-Dawley (Tac:SPRD, outbred stock, mean weight: 293 g, ~8 weeks of age) adult male rats were anaesthetized with s.c. injection of 0.3 ml/100 g fentanyl/fluanisone/midazolam/atropine mixture (2 ml Hypnorm^® ^(fentanyl: 0,315 mg/ml; fluanisone 10 mg/ml), 4 ml midazolam (5 mg/ml), 1.5 ml atropine (1 mg/ml)). Hydrocephalus was induced in a total of thirty-three rats through percutaneous injection into the cisterna magna (25G butterfly needle) of 0.050 ml of sterile kaolin suspension (0.250 mg/mL Ringer's lactate solution (1.4 mM Ca^2+^, 4 mM K^+^, 130 mM Na^+^, 109 mM Cl^-^, 28 mM lactate), H:S pharmacy, Copenhagen University Hospital, Rigshospitalet, Copenhagen, Denmark). The injection was performed after aspiration of CSF through the needle, confirming intracisternal access. Our model had an induction rate of 90% and mortality rate of 9%, corresponding to recently reported rates [20], and better than historically reported rates.

The surviving thirty rats were randomized to 3 groups with different observation periods (2 days (n = 10), 1 week (n = 10), 2 weeks (n = 10)). Eleven control rats received an injection of 0.050 ml vehicle [21,22], and the surviving control rats (n = 10) were divided into approximately equal groups (n = 3/n = 4/n = 3) and assigned to three groups with the same observation periods as kaolin-injected animals. All rats were closely observed throughout recovery from anesthesia, and housed in a controlled environment (~24°C, ~35% humidity, 12 h/12 h light/dark schedule) with *ad libitum *access to food and water. Prior to MRI investigation the rats were weighed and scored for general clinical condition [23,24] daily.

### MR-imaging

At the end of the observation period, MR-imaging was performed using a Varian 4.7T imaging and spectroscopy system. The hydrocephalic rats (two days, n = 10; one week, n = 10; two weeks, n = 10) and the controls (n = 10) were anaesthetized using fentanyl/fluanisone/midazolam/atropine mixture (see above). A tail vein was cannulated and the animal placed inside a home-built 4 cm diameter quadrature head coil. The animal was secured using a stereotactic device incorporated into the design of the head coil. For positioning, a midsaggital scout was acquired before the three main image sequences were performed. For lateral ventricular volume measurements, an optimized T2-weighted fast spin-echo sequence was used. Twenty-two contiguous axial slices (TR = 5600 ms, echo train length = 16, effective TE = 112 ms, NEX = 16, FOV = 35 × 45 mm^2^, matrix 256 × 256, slice thickness = 0.5 mm) were acquired in interleaved order. Image processing software (MIPAV application, http://mipav.cit.nih.gov) was used to delineate the lateral ventricles on the twenty-two slices (Figure 1A, B, C). An approximate value for the volume of the lateral ventricles was then calculated.

In order to determine the water diffusion properties of the tissue, a quantitative diffusion imaging sequence was used. The mid-sagittal scout was used to plan the position of the slices, ensuring that the anatomical sampling in each rat was essentially identical. Twelve contiguous coronal slices were acquired in interleaved order using the following parameters: TR = 1500 ms, TE = 65 ms, FOV = 35 × 35 mm^2^, matrix = 128 × 128, slice thickness = 1 mm, gradient pulse duration δ = 7 ms, gradient pulse separation Δ = 50 ms, b-values = 0, 602, 1069, 1671 s/mm^2 ^with the bipolar gradient applied in three orthogonal directions. Three different ROIs (cortex, periventricular and periaqueductal) corresponding to regions predetermined through Paxinos and Watson's rat brain atlas [25] were drawn on the b = 0 images using MIPAV (Figure 1D). The value of the apparent diffusion coefficient (ADC) for each ROI was calculated using MatLab^® ^software.

In addition, T_1_-weighted pre- and post-contrast injection (0.5 mmol/kg Magnevist (GdDTPA)) spin-echo images were acquired using the following parameters: TR = 300 ms, TE = 14 ms, NEX = 4, matrix = 128 × 128. Slice positioning was identical to the positioning in the diffusion weighted sequence. BBB integrity was determined by subtraction of pre-contrast images from post-contrast images (Figure 1E-G).

### Tissue dissection and preparation

Within 1 h after MRI all rats were euthanized by decapitation whilst under continued anesthesia. The brain was removed and cooled for 10 min at 4°C in 0.1 M PBS-buffer (pH 7.4; 80 mM Na_2_HPO_4_, 20 mM NaH_2_PO_4_, 100 mM NaCl). The brain was embedded in an acrylic brain matrix then sliced in 1 mm slices. The four slices which covered the lateral ventricles and aqueductal area were separated into two pairs. Tissue samples were dissected from one slice of each pair. The dissected samples corresponded to the predetermined ROIs (see above). Samples were frozen in liquid N_2 _and stored for WB. The other slice from each pair was immersion-fixed in 4% formalin solution for 1 h and then stored in 0.1 M PBS-buffer until IHC and IF were performed. The two immersion-fixed slices from each rat were dehydrated in increasing ethanol concentrations overnight and embedded in paraffin wax.

### Western blotting

The frozen tissue samples were homogenized on ice in 1 mL of dissection buffer (0.3 M sucrose, 25 mM imidazole, 1 mM EDTA) supplemented with protease inhibitors (leupeptin and Pefa-block). The homogenate was centrifuged at 4000 g for 15 min. The supernatants were assayed for protein concentration using a bicinchoninic acid (BCA) protein assay (Thermo Scientific, Pierce Protein Research Products, Slangerup, Denmark, cat. no.: 23235) in a standard spectrophotometer. Hereafter, proteins were solubilized in sample buffer (50 mM Tris HCL pH 6.8, 10% glycerol, 6 mg/mL bromophenol blue and 2.5% sodium dodecyl sulphate, SDS) and the final gel sample was made by adding 30 μg/ml dithiothreitol (DTT) and heating the sample at 65°C for 15 min.

Samples were loaded as 15 μg/lane of total protein on a 12.5% SDS-polyacrylamide gel for electrophoresis (Bio-Rad Laboratories, Copenhagen, Denmark, 12.5% Tris-HCL Criterion gel) and were run on a Bio-Rad Criterion Cell system. Each gel represented a ROI. Samples from the control group were loaded together with samples from one of the three kaolin groups. The proteins were transferred onto a nitrocellulose membrane by electroelution using a Bio-Rad Criterion blotting system. Membranes were blocked for 1 h with 5% skimmed milk in phosphate-buffered saline with Tween (PBS-T, PBS with 0.1% Tween 20, pH 7.5), washed three times in PBS-T for 25 min and incubated overnight with following primary antibodies: rabbit anti-AQP4, diluted 1:1000 (Alomone Labs Ltd., Jerusalem, Israel, cat.no. AQP-004) or rabbit anti-actin diluted 1:1000 (Sigma-Aldrich, Brondby, Denmark, cat.no. A2066). The blots were washed, as above, and incubated at room temperature for 1 h with horseradish peroxidase (HRP) conjugated secondary antibody diluted 1:3000 (Dako A/S, Glostrup, Denmark, cat.no.: P0448). Finally, antibody binding was visualized using an enhanced chemiluminescence (ECL) system (Amersham International, GE Healthcare Europe GmbH, Brondby, Denmark).

### Immunohistochemistry

The paraffin-embedded brain tissue slices were cut into serial 6 μm thick frontal sections, which were dewaxed and rehydrated according to standard methods. All sections were incubated in 0.5% H_2_O_2 _in Tris-buffered saline (TBS)/Nonidet (Sigma-Aldrich, cat. no.: N-6507) for 30 min at room temperature to quench endogenous peroxidase. Sections were incubated with 10% normal goat serum (In Vitro, Fredensborg, Denmark, cat. no.: 04009-1B) in TBS/Nonidet for 30 min at room temperature in order to block non-specific binding. Incubation with primary antibodies was performed overnight at 4°C with rabbit anti-AQP4 diluted 1:200 (Alomone Labs Ltd., cat. no.: AQP-004). The primary antibodies were detected by using biotinylated goat anti-rabbit IgG diluted 1:400 (Sigma-Aldrich, cat. no.: 3275) for 30 min at room temperature followed by streptavidin-biotin-peroxidase complex (StreptABComplex/HRP, DAKO, DK, cat. no.: K377) prepared at manufacturer's recommended dilutions for 30 min at room temperature. The immunoreaction was visualized using 0.015% H_2_O_2 _in 3,3-diaminobenzidine-tetrahydrochloride (DAB) as chromogen for 10 min at room temperature. As negative controls, we performed IHC after above mentioned protocol, omitting either primary or secondary antibodies in parallel to standard procedure.

### Immunofluorescence

The AQP4 expressing cell types were characterized by using double fluorescence staining for AQP4 and cell type markers: glial fibrillary acidic protein (GFAP) for astrocytes and tomato lectin for endothelium and microglia. Sections from five rats in each group were incubated overnight with rabbit anti-AQP4 (as above) simultaneously with rat anti-GFAP diluted 1:100 (InVitrogen, Taastrup, Denmark, cat. no.: 13-0300) or fluorescein labeled *lycopersicon esculentum *(tomato) lectin (Vector/VWR, Rodovre, Denmark, cat. no.: FL-1171) diluted 1:50. The primary anti-AQP4 antibodies were detected by using goat anti-rabbit IgG 1:50 linked with Texas red (TxRED) (Southern Biotech, Birmingham, USA, cat. no.: 4050-07), while the anti-GFAP antibodies were detected by using goat anti-rat IgG linked with fluorescein isothiocyanate (FITC) (Calbiochem, The Merck Group, Nottingham, United Kingdom, cat.no.: 401414) for 30 min at room temperature. Finally, sections were incubated with 4',6-diamidino-2-phenylindole (DAPI) (Invitrogen, cat.no.: D1306) for 5 min in order to obtain a nuclear counterstaining. Sections were embedded in anti-fading mounting medium and kept in darkness at -20°C. A Zeiss Axioplan2 light microscope equipped with a triple band (FITC/TxRED/AMCA) filter was used for the examination and recording of the stained specimens.

### Quantification, analysis and statistics

Densitometry of ECL films was performed using QuantityOne software (Bio-Rad Laboratories). Equal total loading of protein was further confirmed by densitometry of the actin signal. Finally AQP4 density was normalized against the β-actin signal.

A twin Zeiss AxioImager A.1 light microscope with a 40× (NA 0.75) objective was used for the examination of the IHC stained specimens. The tissue sections were described in parallel by two observers (ADS and MJ). The upper lateral wall of the lateral ventricle 2-3 mm posterior to Bregma [25] was chosen for our description of the morphological staining pattern. Before examination, the slides were blinded by an independent colleague. Blinding was removed after examination of all slides.

IF-stained sections (five from each group) were analysed qualitatively regarding co-localisation of GFAP and AQP4, and AQP4 expression in relation to lectin staining, blinded by one observer (ADS). 

Data from three non-hydrocephalic animals (all belonging to the two week group) were omitted from the data analysis. Quantitative data from WB and MRI were analysed by calculating median, interquartile range and total range and presented as box plots. Plotting of frequency distribution with and without transformation did not meet the assumption of a normal distribution. Therefore non-parametric testing was used. Significance between groups was determined using the Kruskal-Wallis' test including post test to compare relevant groups. Results from statistics are presented as median (interquartile range), significance level *p *= 0.05. Plot and statistical analyses were performed using the GraphPad Prism Software Package (version 4.03). As we required that the raw data obtained from all three methods (WB, IHC, IF) were of sufficient quality for reliable data analysis, some samples had to be omitted from each technique. The exact numbers are stated in figures and tables.

## Results

### MR-imaging

#### Lateral ventricular volume

**Figure 2:**
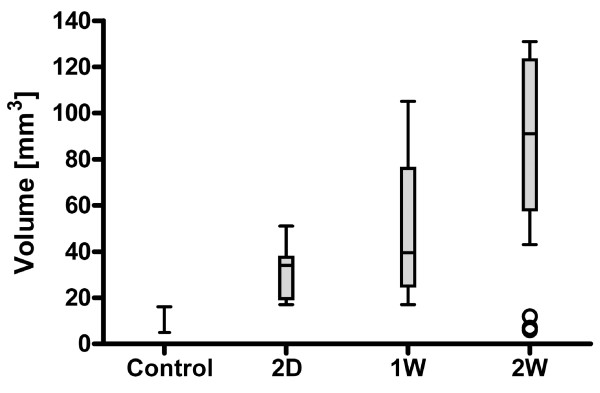
**Lateral ventricular volume**. Quantitative MRI data, presented as box plot (median, interquartiles and range, n = 10 for all groups except 2W when n = 7). In each group the median and interquartile range were as follows (Kruskal-Wallis), control: 5.0 mm^3 ^(4.0-7.5); two days (2D): 34.5 mm^3 ^(19.5-37.5); one week (1W): 39.5 mm^3 ^(25.0-76.0); two weeks (2W): 91.0 mm^3 ^(58.0-123.0). The hydrocephalic groups had significantly larger ventricles, *p *< 0.01, 0.001 and 0.001 at 2 D, 1W and 2W, respectively.

Quantification of lateral ventricular volume by *in vivo *MRI showed failed induction of hydrocephalus in three animals belonging to the two-week group. Consequently, these animals were not analysed resulting in the two-week group consisting of only seven rats. When compared to the lateral ventricles of control animals which was 5.0 mm^3 ^(4.0-7.5, median and interquartile range), significantly enlarged lateral ventricles were observed in hydrocephalic rats at two days, 34.5 mm^3 ^(19.5-37.5), *p *< 0.01; one week, 39.5 mm^3 ^(25.0-76.0) *p *< 0.001; and at two weeks, 91.0 mm^3 ^(58.0-123.0) *p *< 0.001). Results are summarized in Figure 2.

#### Apparent diffusion coefficient

**Figure 3:**
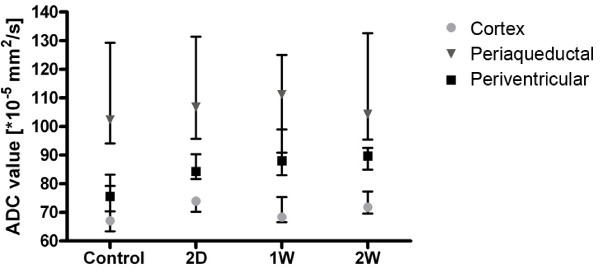
**Apparent diffusion coefficient (ADC) values**. Box plot showing each region of interest in each group (control and 2-day, 1-week and 2-weeks hydrocephalus) quantified by MRI (median, interquartiles and range, n = 10 for all groups except 2W when n = 7). Periventricular ADC values showed a significant difference (Kruskal-Wallis) between the control group and both the one week and two week group, *p *< 0.05 for both. Control: 75.5*10^-5^mm^2^/s (70.4-83.2; two days: 84.3*10^-5^mm^2^/s (81.6-90.4); one week: 88.1*10^-5^mm^2^/s (83.0-99.1); two weeks 89.1*10^-5^mm^2^/s (85.9-90.3). No significant differences were found between groups in any other regions of interest.

ADC values were calculated for each of the ROIs and the results are presented in Figure 3. Significant differences occurred in the periventricular ROI between the ADC values of control and both one-week and two-week groups (Kruskal-Wallis, *p *< 0.05) but not in the 2-day group. Control: 75.5*10^-5^mm^2^/s (70.4-83.2); two days: 84.3*10^-5^mm^2^/s (81.6-90.4); one week: 88.1*10^-5^mm^2^/s (83.0-99.1); two weeks 89.1*10^-5^mm^2^/s (85.9-90.3). No significant differences between control and any group of hydrocephalic rats were observed in the other regions.

#### BBB integrity

The integrity of the BBB was assessed by image subtraction of pre- from post-contrast T1-weighted images (Figure 1E, F, G). Qualitative description showed no signal enhancement besides the expected vessel enhancement. This confirmed preserved BBB integrity in all cases.

### AQP4 expression

#### Western Blotting

**Figure 4:**
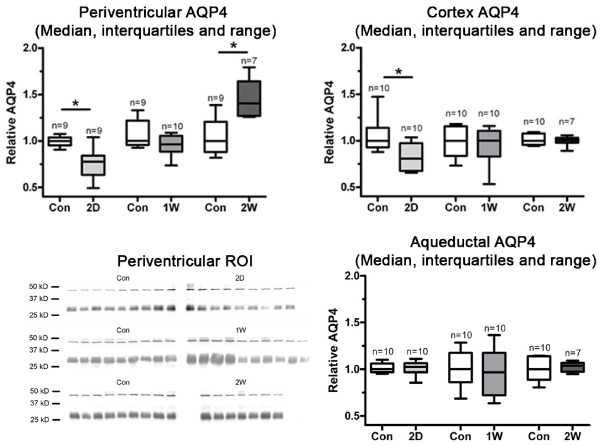
**Aquaporin-4 western blotting**. Immunoreactivity signal at ~42 kD corresponds to β-actin, and ~30-32 kD corresponds to aquaporin-4. Western blots from the periventricular region of interest are shown as example. Two of the periventricular samples (one control and one two day) did not meet the minimum total protein level for western blotting, and therefore data from these two rats could not be obtained. Densitometry of ECL films was performed using QuantityOne software (Bio-Rad Laboratories). Aquaporin-4 signal was normalized against the β-actin signal. Relative aquaporin-4 expression values for each ROI are presented as box plots (median, interquartile range and total range). At day two a significant (Kruskal-Wallis) decrease compared to control in aquaporin-4 expression was found in both periventricular region (control: 1.00, 0.95-1.04, two day: 0.78, 0.63-0.084, *p *< 0.05) and cortex (control: 1.00, 0.93-1.14, two day: 0.81, 0.67-0.97, *p *< 0.05). Expression was normalized after one week, but after two weeks we found significantly increased periventricular expression (control: 1.00, 0.88-1.21; two weeks: 1.40, 1.27-1.64, *p *< 0.05). No significant changes were found in the periaqueductal region.

Western blotting revealed an immunoreactivity signal at ~42 kD corresponding to β-actin, and ~30-32 kD corresponding to AQP4 (Figure 4). AQP4 expression was significantly decreased (Kruskal-Wallis) relative to control in both the periventricular region after two days of hydrocephalus (control: 1.00, 0.95-1.04, two day: 0.78, 0.63-0.084, *p *< 0.05) and in the cortex (control: 1.00, 0.93-1.14, two day: 0.81, 0.67-0.97, *p *< 0.05) and normalized after one week. After two weeks significantly increased periventricular AQP4 expression was observed (control: 1.00, 0.88-1.2; two weeks: 1.40, 1.27-1.64, *p *< 0.05). No change in AQP4 expression was found in the periaqueductal region at any time.

#### Immunohistochemistry

**Figure 5:**
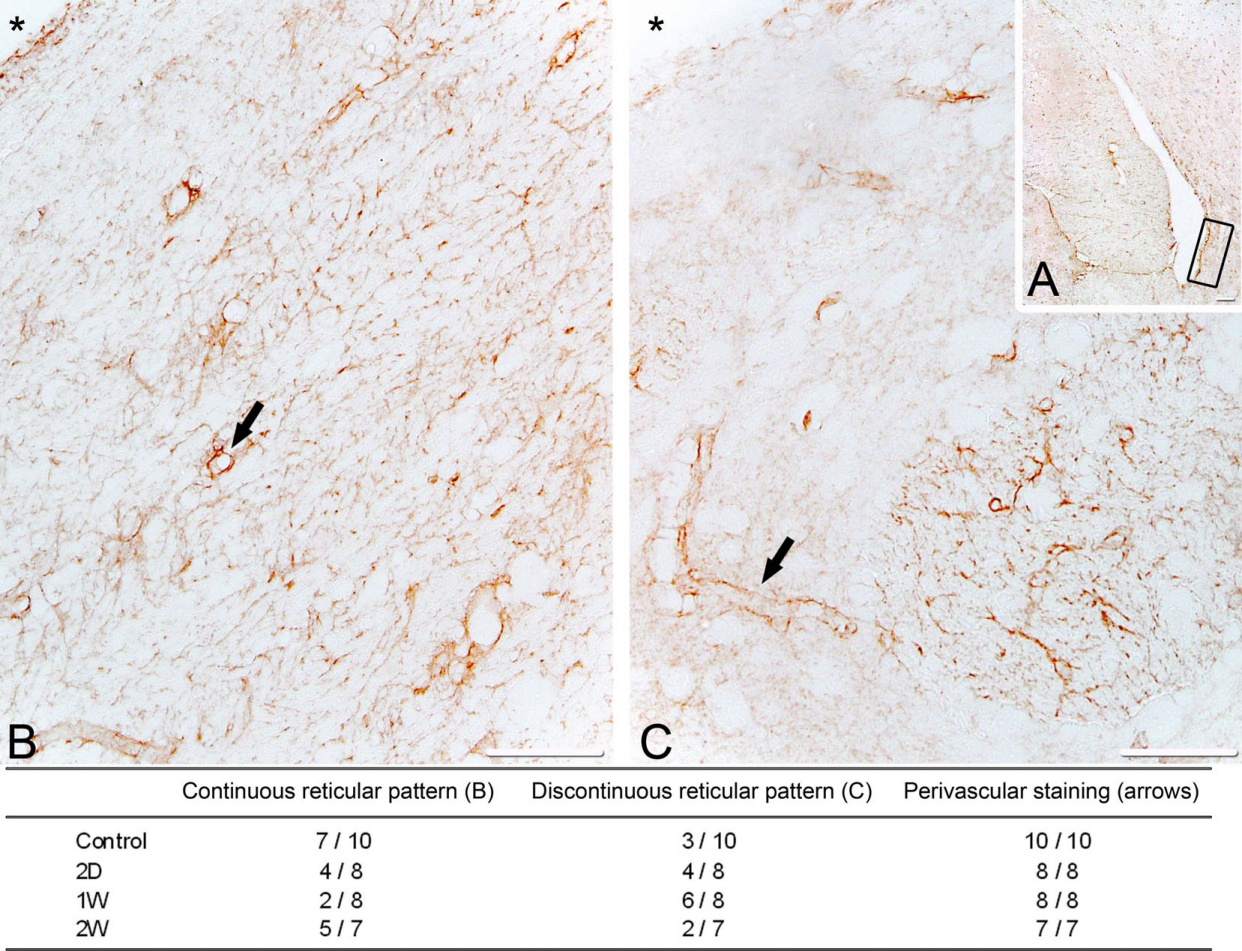
**Immunohistochemistry of aquaporin-4 in the periventricular region**. A: low magnification picture representing a control rat. The boxed area shows the studied periventricular region. B and C: different patterns of aquaporin-4 immunoreactivity. Aquaporin-4 positive glial processes were identified as reticular pattern and perivascular "vessel-like" structures. "Vessel-like" immunoreactivity was identified either as strongly labeled round/eliptical structures where we expect a vessel is cut across (B, arrow) or as parallel lines with branches where a vessel is cut in its length direction (C, arrow). "Vessel-like" immunoractivity was identified in all cases. The reticular pattern could further be categorized as either continuous (B) or discontinuous (C). In cases of discontinuous reticular pattern, larger areas were found devoid of the reticular pattern. In controls the continuous pattern prevailed, in the acute phase (two day and one week) a trend pointed towards the discontinuous pattern, and finally at 2 weeks the reticular pattern was again prominent. The inserted table summarizes the proportion of rats in each group presenting either continuous or discontinuous reticular pattern and the proportion of rats showing perivascular "vessel-like" immunoreactivity. This suggests a dynamic response in the expression pattern of aquaporin-4. No evidence of immunoreactivity in cell soma of astrocytes was found. Negative immunohistochemistry controls did not show any immunoreactivity. * = lateral ventricle. A scale bar 100 μm; B and C scale bar 50 μm.

**Figure 6:**
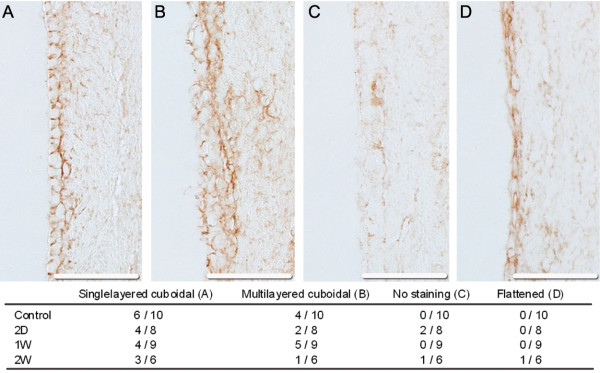
**Aquaporin-4 immunohistochemistry of the ependyma**. The aquaporin-4 positive ependymal cell lining of each rat was categorized as belonging to one of four different aquaporin-4 immunoreactivity morphologies (single-layered cuboidal (A), multilayered cuboidal (B), no staining (C) and flattened ependyma (D)). The table shows the proportion of animals in each group belonging to each morphological category. Cuboidal ependyma in single- or multilayered patterns were found in all groups. In the two day group, two cases were found to have lost ependymal staining. At two weeks a diversity of morphologies were reported ranging from single layered cuboidal to flattened in the case of most extreme hydrocephalus. No clear evidence of apical expression of aquaporin-4 in ependymal cells was found at any time point. Scale bar 50 μm.

Intraparenchymal AQP4 immunoreactivity in the periventricular region consistently showed a reticular staining pattern indicating that AQP4 reactivity occurred in glial processes (Figure 5). The continuous glial reticular pattern seemed to diminish in the acute (2 days) and subacute (1 week) phases of hydrocephalus and re-appear in the later stages (2 weeks), possibly reflecting the time-dependent quantitative changes observed by WB. Immunoreactivity of the ependyma showed a cuboidal pattern in all controls appearing either single- or multilayered [26]. In hydrocephalic animals, the ependymal pattern was disrupted in several cases; particularly in the 2-week group. In this group, several types of ependymal changes could be seen including loss of ependymal staining, flattening or a relative loss of multilayering (Figure 6).

No difference in AQP4 immunoreactivity of glia limitans or in the periaqueductal region was identified in hydrocephalic groups compared to controls. No AQP4 immunoreactivity of choroid plexus was seen. The specificity of the immune reaction was confirmed by the presence of perivascular "vessel-like" staining identified in all cases by the negative control sections, which were devoid of AQP4 immunostaining.

#### Double fluorescence staining

**Figure 7:**
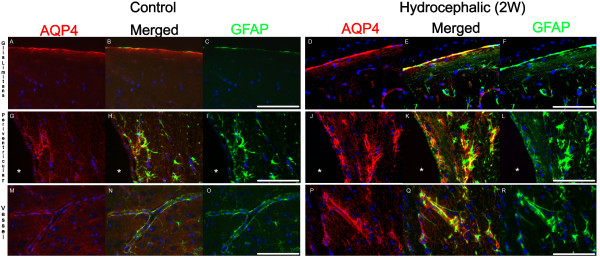
**Double immunofluorescence for aquaporin-4 and glial fibrillary acidic protein in control and two week hydrocephalic rat**. Aquaporin-4 is visualized with TxRED (red) and glial fibrillary acidic protein with FITC (green). Nuclear counterstaining with DAPI (blue). Pictures represent glia limitans (A-F), periventricular region (including ependyma) (G-L) and vessels (M-R). Yellow colours in merged pictures confirm co-localization of aquaporins-4 and astrocytic GFAP in glia limitans and in perivascular endfeet. Both in control and in all hydrocephalic groups aquaporin-4 and glial fibrillary acidic protein showed co-localisation in the astroglial endfeet surrounding the vessels (M-R) and in glia limitans (A-F). Astrocytic cell soma were mainly without evidence of aquaporin-4 expression. * = lateral ventricle. Scale bar 100 μm.

**Figure 8:**
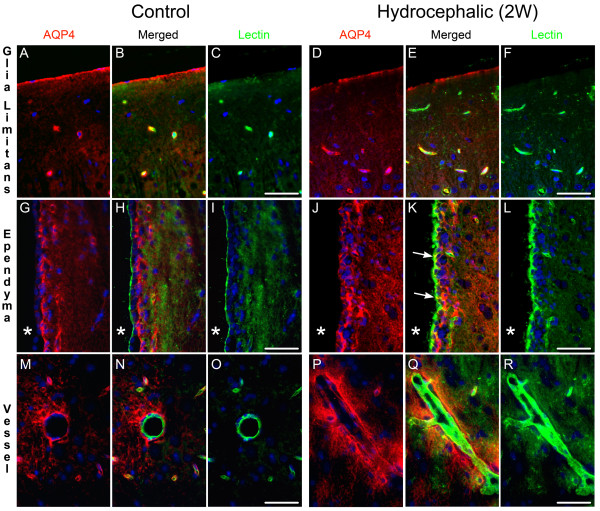
**Combined aquaporin-4 immunofluorescence and flouorescein linked lectin staining in control and two week hydrocephalic rat**. Aquaporin-4 visualized with TxRED (red) and lectin with FITC (green). Nuclear counterstaining with DAPI (blue). Pictures represent glia limitans (A-F), ependyma (G-L) and vessels (M-R). In both contol and hydrocephalic rats aquaporin-4 and lectin staining showed complementary immunoreactivity, clearly not co-localized in glia limitans (A-F). In the ependyma lectin intensively stained the apical membrane of the cells bordering the ventricular wall (H, I, K, L). Immunoreactivity of aquaporin-4 in controls clearly localised to the basolateral membrane (G). In hydrocephalic animals presenting changed aquaporin-4 morphology of the ependyma (J-L), the aquaporin-4 immunoreactivity of the cells lining the ventricle was found not to co-localize with the continuous apical staining of the ependymal cells by lectin (K), except in specific areas corresponding to the lateral membrane of the ependymal cells (arrows, K). This confirmed basolateral expression of aquaporin-4 in the ependymal cells of hydrocephalic rats. In vessels (M-R) aquaporin-4 was clearly located to perivascular areas without signs of endothelial expression. No evidence of aquaporin-4 expression in microglia was found; neither in controls nor in hydrocephalic rats. * = lateral ventricle. Scale bar 50 μm.

Fluorescence staining for AQP4 and GFAP or lectin revealed that astroglia are the main source of AQP4 expression in the brain, while endothelial cells and microglia are devoid of AQP4 expression (Figure 7 and Figure 8). Double IF for AQP4 and GFAP showed co-localisation both in control and hydrocephalic groups at all time points, in which AQP4 was observed in the astroglial endfeet surrounding the vessels and in glia limitans (Figure 7). Combined AQP4 IF and lectin fluorescence staining in both controls and hydrocephalic rats showed complementary immunoreactivity, clearly not co-localized in glia limitans or in endothelial cells (Figure 8). In both controls and hydrocephalic rats microglia stained by lectin did not express AQP4. Lectin in all cases intensively stained the apical membrane of the ependymal cells. Immunoreactivity of AQP4 both in controls and hydrocephalic rats localised to the basolateral membrane of the ependymal cells lining the ventricular wall, thus confirming conserved polarisation of the AQP4 positive ependymal cells in hydrocephalus.

### Correlation between periventricular AQP4 expression and quantitative MRI

**Figure 9:**
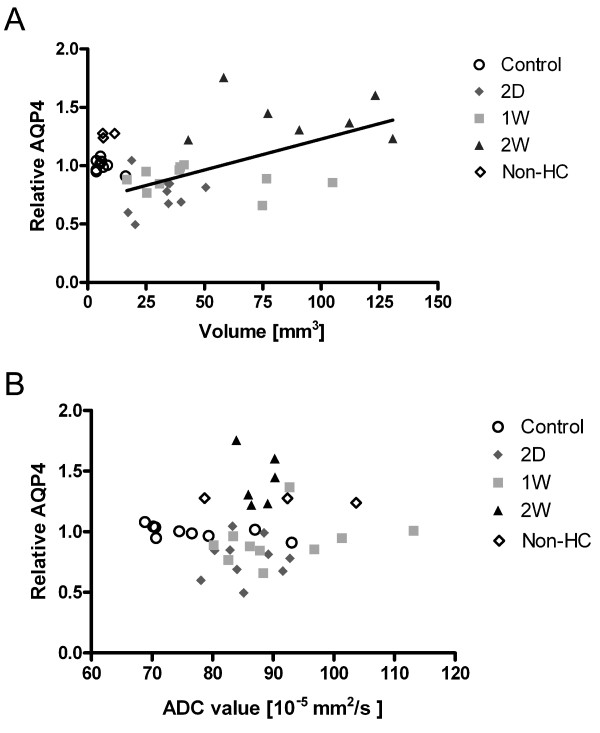
**Correlation between quantitative MRI and relative AQP4 expression**. Plots of lateral ventricular volume vs. relative aquaporin-4 expression (A) and periventricular apparent diffusion coefficient value vs. relative aquaporin-4 expression (B) in both controls, hydrocephalic (two days, 2 D, one week,1W and two weeks, 2W) and non-hydrocephalic kaolin-injected animals. In hydrocephalic animals we found a significant linear positive correlation between lateral ventricular volume and periventricular aquaporin-4 expression (r^2 ^= 0.33, p = 0.002). No correlation between periventricular apparent diffusion coefficient value and relative aquaporin-4 expression was found.

In order to test the hypothesis that AQP4 expression could be related to severity of hydrocephalus, we performed linear regression analysis of AQP4 expression relative to median of control group against lateral ventricular volume across all groups (Figure 9). We found a significant linear positive correlation between lateral ventricular volume and periventricular AQP4 expression (r^2 ^= 0.33, *p *= 0.002) in hydrocephalic animals. No significant linear correlation between periventricular AQP4 expression and ADC value was found (Figure 9).

## Discussion

The present study shows regional- and time-dependent regulation of AQP4 expression in experimental hydrocephalus. In summary, AQP4 abundance was found significantly decreased relative to control in both the periventricular region and in the cortex after two days of hydrocephalus and normalized after one week. After two weeks of hydrocephalus significantly increased periventricular AQP4 expression was observed. The changes in AQP4 expression coincided with significantly increased periventricular ADC value after one and two weeks of hydrocephalus, but no direct linear correlation was found. Double fluorescence staining confirmed the cell-specific expression of AQP4 and did not provide any evidence of endothelial AQP4 expression in hydrocephalus. Previously, only a few studies have investigated AQP4 expression in hydrocephalus (recently reviewed by Owler *et al *[27] and Filippidis *et al *[28]). In a hydrocephalus model in adult rats induced by L-α-lysophosphatidylcholine stearoyl (LPS), ventriculomegaly was correlated to increased AQP4 in astrocytes residing in periventricular areas such as in corpus callosum [17]. In kaolin-mediated severe hydrocephalus in neonatal (one-day old) and juvenile (three weeks old) rats, an intense cerebral, perivascular AQP4 immunostaining was reported at four weeks and nine months post-injection in the juvenile animals, although the study did not find significant AQP4 changes by using WB [18]. However, the finding of AQP4 immunoreactivity in perivascular astrocytes in LPS-induced hydrocephalus [17] support the data presented in this study showing time-dependent AQP4 changes following kaolin injection. The observed increase in AQP4 levels seen at 2 weeks in this study are likely due to *de novo *AQP4 synthesis, as AQP4 mRNA is significantly upregulated in hydrocephalic rat brains [18]. In an experimental study of congenital hydrocephalus [19] using the H-Tx rat [29] it was suggested that AQP4 could be important for developing alternative CSF-absorption pathways [16].

The functional significance of AQP4 during hydrocephalus has previously been investigated by using AQP4-null mice that show accelerated progression of ventriculomegaly relative to wild type controls [15]. This suggests that cerebral AQP4 is neuroprotective and accordingly, the increase in AQP4 expression as presented here might represent a host defense mechanism against kaolin-mediated pathology. A similar role of AQP4 is reported in a bacterial brain abscess coupled with vasogenic edema, in which AQP4-deficient mice show significantly higher ICP, brain swelling, and cerebral water content than their wild type controls [30]. Also, in 3 different models of brain edema such as a freeze-injury model of cerebral vasogenic edema [31], a brain tumor model of edema [31] and a model of edema following subarachnoid haemorrhage (SAH) [32], AQP4-deficient mice displayed increased CNS water accumulation, deteriorated neurological outcome and increased ICP when compared to those of wild type control mice [31-33]. Hydrocephalic edema however represents water accumulation different from both vasogenic and cytotoxic edema, because of the absence of BBB disruption and cell swelling. A model of continuous intracerebral fluid infusion mimicking purely interstitial edema showed a poorer outcome in AQP4-null mice [31]. AQP4 deficient animals thus have a poorer outcome in conditions with both vasogenic and interstitial (hydrocephalic) edema. All these data indicate a role for AQP4 in the cerebral resolution of an established edema, in which the mechanisms likely involve astroglial clearance of excess brain water by transcellular routes and/or through the glia limitans. However, contrasting data are found in studies of other CNS disease models (such as ischemia, meningitis and trauma), in which AQP4-null mice display a better outcome, in terms of neurological score and brain water content, than wild type controls [34-36]. These studies indicate that altered AQP4 expression is rate-limiting for brain water transport and edema implying that altered AQP4 expression maybe functionally significant in cases of disturbed CSF circulation. Specifically, the results obtained in AQP4-deficient mice along with our data suggest that the dual regulation of AQP4 could serve as a compensatory host defense response to obstructive hydrocephalus. We therefore suggest a biphasic neuroprotective response consisting of down-regulation of AQP4 after two days, corresponding to acutely increased ICP, and up-regulation after two weeks, corresponding to a near-normal ICP phase [15,37-40]. The phenotypic and pathological analyses of AQP4-modulated mice indicate a role for AQP4 in cerebral bidirectional water transport and edemal pathophysiology. As discussed here, cerebral AQP4 might exert a paradoxic role contributing to cytotoxic/cellular edema while protecting against vasogenic/interstitial edema in the brain. Thus, AQP4 inhibition or antagonism of its signalling, potentially reduce the development of cytotoxic edema by means of reducing cellular water accumulation in the brain. In contrast, AQP4 mimetics or inducers of AQP4 expression in astroglia can likely remove excess brain water from the extracellular compartment thereby facilitating the resolution of vasogenic/interstitial edema. However, the molecular mechanisms of actions and/or the signalling pathways involved in the differential effects of AQP4 in the intracellular versus extracellular space, respectively, remain to be elucidated. Yet, current data support the notion of AQP4 exerting such dual roles that likely are functionally separated by means of the spatial compartment (inside vs. outside cells) and differential molecular surroundings found in the extracellular vs. intracellular space. Supporting this notion, potassium dynamics within cerebral extracellular matrix and the brain's extracellular tissue volume are closely associated to AQP4 protein levels [41,42]. We hypothesize that pharmaceutical regulation of AQP4 may be used in the future to manage the clinical course and neurological outcome of hydrocephalus. The potential effects of pharmacological intervention may not be limited to the water transport role of AQP4 since recent research suggests additional functions. Evidence from *in vitro *studies propose that AQP4 acts as a cell adhesion molecule [43,44], maintains ependymal integrity [45] and plays a significant role in adult neurogenesis [46,47]. One study [48] connected the different functions of AQP4 to hydrocephalus by reporting a 9.6% rate of sporadic obstructive hydrocephalus as consequence of disorganised ependyma and aqueductal stenosis in AQP4-null mice. Whilst our data also suggest disorganisation of the ependyma as well as changes in AQP4 expression, our study design does not allow us to address any specific correlation. Further studies therefore need to consider that pharmacological regulation of AQP4 may influence hydrocephalus in different ways.

Our data confirm astrocytic and ependymal cell localization of AQP4 in both normal brain and in hydrocephalic brain pathology. AQP4 co-localizes with astrocytic GFAP in both glia limitans and perivascular end feet. These results support original studies by IHC and high-resolution electron microscopy on cellular domains expressing AQP4 in rat brain under normal conditions [10,14,49]. The studies revealed polarised expression of AQP4 under normal physiological conditions. AQP4 was restricted to glial cells with morphologic features typical of astrocytes and ependymal cells. The most distinct expression was found in glial membranes facing capillaries and pia mater [10,14,49]. We saw no evidence of apical AQP4 expression in ependymal cells at any time point, providing no evidence for the "missing step" in water transport across the ependymal cell membrane. By double fluorescence combining AQP4 and lectin staining, we found no evidence of AQP4 expression in microglia. In contrast AQP4 mRNA and protein has been reported in reactive OX-6 positive microglia after LPS injection [50]. No evidence for neuronal expression of AQP4 currently exists [10,14,51,52]. In vascular structures, AQP4 co-localized to astroglia but not to endothelial cells. One study [53] arguments for endothelial expression of AQP4 mRNA and protein in preparations of rat cerebral microvessels. This contrasts to our and other's previous data [14]. It is possible, as the authors suggest themselves, that the preparations were contaminated with glial membranes. Thus, our data do not suggest changed cell type expression of AQP4 in hydrocephalus, but rather changed level of the constitutive expression.

Our observation of decreased abundance of AQP4 in the periventricular region after two days of hydrocephalus coincide with symptoms indicative of raised intracranial pressure (ICP) as judged by clinical symptoms. This is further supported by the time course of ICP in kaolin-mediated hydrocephalus, where an acute rise in ICP is seen within one day followed by a markedly elevated but stationary ICP that after several weeks ultimately approached a near-normal level [15,37-40]. Together with the acute decreased abundance of AQP4 in the periventricular region we found normal ADC-values in the same region. At two weeks, increased abundance of AQP4 expressed in astrocyte processes of the periventricular region was found, together with significant elevated ADC values. Linear regression did not propose direct linear correlation of ADC values and AQP4 expression in the periventricular region, in opposition to data presented in one other study [17]. We consider that changes in ADC values and AQP4 represent phases of ICP changes following hydrocephalus development. Other authors present similar initially non-significant decreases in periventricular white matter ADC values one day after kaolin injection followed by significant increases in periventricular ADC at eight days [54]. Caution should be taken when interpreting ADC values. By using diffusion weighted MRI and calculating ADC values it is possible to identify acute CNS injury following e.g. ischemia and status epilepticus [54-56]. It has been proposed that decreased ADC values may result from cell swelling, loss of extracellular volume and reduction in transmembrane water movement [54]. In the same study it was shown that cell injury following global ischemia causes decreased ADC values in both extra- and intracellular compartments [54]. Therefore, ADC value alone cannot distinguish between intracellular and extracellular edema. In hydrocephalus the periventricular extracellular volume is increased either by direct passage of CSF from the lateral ventricles or by stagnant periventricular fluid generated from brain tissue [57]. We believe that the changes in ADC values observed in this study are indicative of extracellular edema. The missing correlation between AQP4 expression and ADC-values indicate that other factors such as co-transporters may play a major role in brain water transport.

## Conclusions

This study provides evidence of significant variation in the temporal and spatial regulation of AQP4 in the kaolin-induced hydrocephalic rat brain. Novel data confirm a dynamic response consisting of significantly decreased abundance of AQP4 in the periventricular region and cortex at two days and significantly increased periventricular abundance of AQP4 after two weeks of hydrocephalus. In addition we present data confirming an astrocytic and ependymal origin of AQP4 expression during hydrocephalic brain pathology. ADC values show the presence of periventricular interstitial edema at one and two weeks of hydrocephalus. In the light of the putative differential roles of AQP4 discussed in this study, this could point towards a role for AQP4 as a drug target with the potential to be clinically exploited.

## List of abbreviations

ADC: apparent diffusion coefficient; AQP: aquaporin; BBB: blood brain barrier; CNS: central nervous system; CSF: cerebrospinal fluid; DAPI: 4',6-diamidino-2-phenylindole; FITC: fluorescein isothiocyanate; GFAP: glial fibrillary acidic protein; ICP: intracranial pressure; IF: immunofluorescence; IHC: immunohistochemistry; LPS: L-α-lysophosphatidylcholine stearoyl; MRI: magnetic resonance imaging; ROI: region of interest; SAH: subarachnoid haemorrhage; SAS: subarachnoid space; TxRED: Texas red; WB: western blotting.

## Competing interests

The authors of this paper declare they have no actual or potential conflict of interest nor financial, personal, contractual or other relationships with other people or organizations that could inappropriately influence the work.

## Authors' contributions

ADS conceived of the study, participated in its design and coordination, performed practical animal handling, MRI data analysis, WB lab work, WB data analysis, IHC lab work, IHC data analysis, IF lab work, IF data analysis and drafted the manuscript. IJR performed practical animal handling, MRI sequence development, MRI data analysis and drafted the manuscript. LVS performed MRI sequence development, MRI data analysis and drafted the manuscript. JP conceived of the study, participated in its design and coordination, performed WB lab work, WB data analysis, IHC data analysis and drafted the manuscript. MP performed IHC lab work, IF lab work and drafted the manuscript. MJ conceived of the study, participated in its design and coordination, performed IHC data analysis and drafted the manuscript. All authors have read and approved the final version of the manuscript.
